# Use of Recombinant Endolysin to Improve Accuracy of Group B Streptococcus Tests

**DOI:** 10.1128/spectrum.00077-21

**Published:** 2021-08-11

**Authors:** Hidehito Matsui, Jumpei Uchiyama, Masaya Ogata, Tadahiro Nasukawa, Iyo Takemura-Uchiyama, Shin-ichiro Kato, Hironobu Murakami, Masato Higashide, Hideaki Hanaki

**Affiliations:** a Ōmura Satoshi Memorial Institute, Kitasato University, Tokyo, Japan; b Department of Bacteriology, Graduate School of Medicine Dentistry and Pharmaceutical Sciences, Okayama University, Okayama, Japan; c School of Veterinary Medicine, Azabu University, Sagamihara, Kanagawa, Japan; d Kochi University, Kochi, Japan; e Kotobiken Medical Laboratories, Inc., Tsukuba, Ibaraki, Japan; University of Cincinnati

**Keywords:** endolysin, group B streptococcus, *Enterococcus faecalis*, *Streptococcus agalactiae*, GBS culture test

## Abstract

Group B Streptococcus (GBS) causes serious neonatal infection via vertical transmission. The prenatal GBS screening test is performed at the late stage of pregnancy to avoid risks of infection. In this test, enrichment culture is performed, followed by GBS identification. Selective medium is used for the enrichment; however, Enterococcus faecalis, which is a potential contaminant in swab samples, can interfere with the growth of GBS. Such bacterial contamination can lead to false-negative results. Endolysin, a bacteriophage-derived enzyme, degrades peptidoglycan in the bacterial cell wall; it is a promising antimicrobial agent for selectively eliminating specific bacterial genera/species. In this study, we used the recombinant endolysin EG-LYS, which is specific to E. faecalis; the endolysin potentially enriched GBS in the selective culture. First, in the false-negative model (coculture of GBS and E. faecalis, which disabled GBS detection in the subsequent GBS identification test), EG-LYS treatment at 0.1 mg/ml improved GBS detection. Next, we used 548 vaginal swabs to test the efficacy of EG-LYS treatment in improving GBS detection. EG-LYS treatment (0.1 mg/ml) increased the GBS-positive ratio to 17.9%, compared to 15.7% in the control (phosphate-buffered saline [PBS] treatment). In addition, there were an increased number of GBS colonies under EG-LYS treatment in some samples. The results were supported by the microbiota analysis of the enriched cultures. In conclusion, EG-LYS treatment of the enrichment culture potentially improves the accuracy of the prenatal GBS screening test.

**IMPORTANCE** Endolysin is a bacteriophage-derived enzyme that degrades the peptidoglycan in the cell wall of host bacteria; it could be used as an antimicrobial agent for selectively eliminating specific bacterial genera/species. Group B Streptococcus (GBS) causes neonatal infection via vertical transmission; prenatal GBS screening test, in which enrichment culture is followed by bacterial identification, is used to detect the presence of GBS in pregnant women. However, the presence of commensal bacteria such as Enterococcus faecalis in clinical specimens can inhibit GBS growth in the selective enrichment culture, resulting in false-negative result. Here, we demonstrated that the application of originally isolated endolysin in the enrichment culture improved the test accuracy by inhibiting unwanted E. faecalis growth and therefore avoiding false-negative results, not only in experimental settings, but also in tests using vaginal swabs.

## INTRODUCTION

Streptococcus agalactiae or group B Streptococcus (GBS) is often transmitted vertically from pregnant women to newborns during delivery, potentially leading to fatal neonatal infections such as sepsis, pneumonia, or meningitis (1, 2). To avoid the risk of infection, a GBS culture test is often performed during the late stage of pregnancy (1, 2), and prophylactic antibiotics are prescribed to GBS-positive pregnant women (1, 2). In the prenatal GBS screening test, the enrichment culture of a vaginal and anorectal swab is followed by a bacterial identification test to improve GBS detection (3, 4). The GBS identification test is carried out using culture-based and/or nucleic acid amplification tests (4). The selective culture media used for GBS enrichment, such as Granada broth and Lim broth, are formulated to minimize the growth of bacteria other than GBS (5, 6). However, Enterococcus faecalis growth in GBS cultures disturbs the growth of GBS, possibly leading to false-negative results (7–9). The culture-based bacterial identification tests, following enrichment culture, have a false-negative rate of approximately 3 to 7% (10, 11). To reduce cases of GBS infection in neonates, it is important to improve the test accuracy by inhibiting the growth of E. faecalis in the enrichment culture.

Bacteriophages (phages) specifically infect bacterial cells through compatible receptors or growth-supporting systems. Most phages are highly host-specific, infecting only a particular species or genera (12). Once phages infect the host bacterial cells, they propagate within the cells and release progeny phages into the milieu. In the late stage of phage infection, endolysin, a peptidoglycan-hydrolyzing enzyme encoded by the phage genome, plays a crucial role in the hydrolysis of the peptidoglycan network, facilitating the release of progeny phages (13, 14). The specificity of endolysin is limited to specific bacterial genera and/or species; however, it generally has broader specificity than phages because it is not influenced by bacterial host immunity (15, 16). Phages and endolysin could be used to inhibit unwanted E. faecalis growth.

We previously reported the use of an E. faecalis-specific phage cocktail as an E. faecalis selective growth inhibitor in the prenatal GBS screening test (17). However, the introduction of this phage cocktail for clinical use in prenatal GBS screening tests could be limited by the following factors: (i) phages in the mixture must be monitored consistently because phage-insensitive or -resistant bacteria could emerge (18); (ii) the phage cocktail needs to be optimized, as the phages in the cocktail may interfere with each other (19); (iii) it may take longer than expected to prepare the phage cocktail, because the process for manufacturing each phage requires optimization (20); and (iv) the use of phages in clinical laboratories may cause phage contamination in clinical specimens (17).

The use of endolysin is considered to overcome these issues and has the following advantages over phage cocktail in the prenatal GBS screening test: (i) a single endolysin alone is likely to cover a broader range of target bacterial strains than a single phage, and therefore the process development of endolysin manufacturing requires less effort than that for the phage cocktail (13, 21); (ii) emergence of endolysin-resistant mutants is more unlikely than the emergence of phage-resistant bacteria (13); and (iii) recombinant endolysin is commercially available for therapeutic purposes (22), and therefore the exploration of its use in prenatal GBS screening test saves time and money.

We hypothesized that the use of endolysin could improve the prenatal GBS screening test by reducing the number of false-negative results caused by E. faecalis in the enrichment culture. In this study, we tested the potential of endolysin in the enrichment culture followed by culture-based identification tests for reducing the number of false-negative results by suppressing E. faecalis growth in the prenatal GBS screening test using clinical specimens.

## RESULTS AND DISCUSSION

### Recombinant endolysin of phage phiM1EF2.

We isolated the E. faecalis phage phiM1EF2, which belongs to the family Siphoviridae and genus Saphexavirus, based on its genome sequence similarity to phages belonging to this family. Open reading frame 60 (ORF60) consisted of 237 amino acids containing the CHAP domain, which was estimated to be 26.1 kDa. ORF60 is an amidase, and therefore it was predicted to be an endolysin. Recombinant ORF60 fused with a 6×His tag at the C-terminal end was produced in Escherichia coli and purified using affinity chromatography (see Fig. S1A in the supplemental material). The enzymatic activity was tested spectrophotometrically using peptidoglycan extracted from E. faecalis KUEF29 as the substrate in phosphate-buffered saline (PBS; pH 7.4). The enzyme decreased the turbidity in a dose- and time-dependent manner (see Fig. S1B in the supplemental material). We tentatively designated the purified recombinant protein ORF60 as EG-LYS.

**FIG 1:**
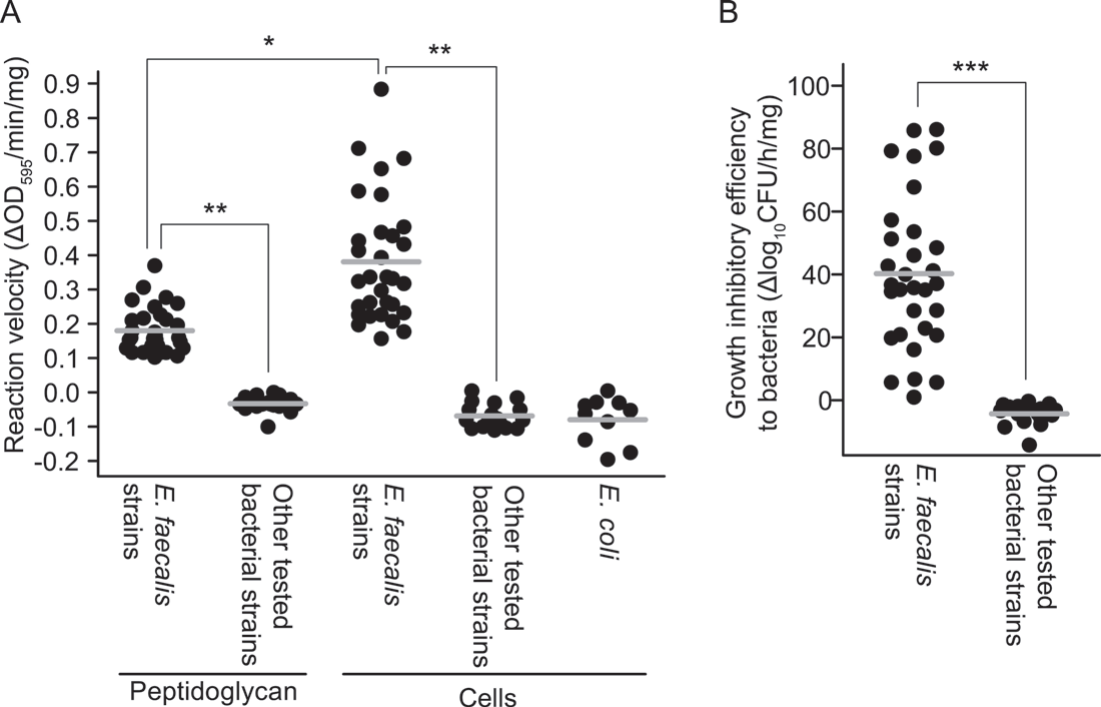
EG-LYS activity *in vitro*. (A) Reaction velocities of EG-LYS on peptidoglycan and bacterial cells. The reaction velocity in change of optical density at 595 nm (ΔOD_595_)/min/mg was calculated from the difference in turbidity change between EG-LYS and phosphate-buffered saline (PBS) treatments divided by time and the amount of EG-LYS used (see Fig. S6 in the supplemental material). Each dot represents the velocity of EG-LYS on peptidoglycan or bacterial cell and is an average of triplicate data. Escherichia coli strain DH5α was used as a negative control, and the experiments were repeated 10 times. The mean value of the velocities of EG-LYS treatment is shown by a thick gray line. The data were analyzed using the Kruskal-Wallis test followed by *post hoc* Dunn’s multiple-comparison test. Statistical significances at *P < *0.05 and *P < *0.001 are shown as single (*) and double (**) asterisks, respectively. (B) Growth-inhibitory efficiencies of EG-LYS treatment against intact bacterial cells. The growth-inhibitory efficiency in Δlog_10_CFU/h/mg was calculated from the difference of bacterial growth between EG-LYS and PBS treatments divided by incubation time and the amount of EG-LYS used (see Fig. S7 in the supplemental material). Each dot represents the growth inhibitory efficiency of an individual strain. The mean value is shown as a bold gray line. The data were analyzed using the Mann-Whitney U test. Statistical significance at *P < *0.0001 is denoted by a triple asterisk (***).

EG-LYS activity was examined using E. faecalis, Enterococcus faecium, Enterococcus avium, and GBS strains isolated from the vaginal swabs in the prenatal GBS screening test (17). First, EG-LYS activity on peptidoglycan and whole bacterial cells in PBS was analyzed. The reaction velocity of EG-LYS on peptidoglycan and whole bacteria (change in optical density at 595m nm [ΔOD_595_]/min/mg protein) was examined, and it was calculated as the difference in turbidity reduction between EG-LYS and PBS treatments divided by the incubation period and the amount of EG-LYS used (Fig. 1A). The reaction velocities of EG-LYS on Escherichia coli DH5α cells were used as negative controls (mean ± standard deviation [SD]; −0.0798 ± 0.0675 ΔOD_595_/min/mg). The reaction velocities of EG-LYS on the peptidoglycan and intact cells of E. faecalis (0.1801 ± 0.0658 and 0.3808 ± 0.1798 ΔOD_595_/min/mg, respectively) were significantly higher than that in the negative control. However, the reaction velocities of EG-LYS on the peptidoglycan and intact cells of E. faecium, *E. avium*, and GBS were nearly indistinguishable from that in the negative control.

Moreover, we examined the growth-inhibitory efficacy of EG-LYS treatment (Δlog_10_CFU/h/mg), which was calculated as the difference in bacterial growth between EG-LYS and PBS treatments divided by the incubation period and amount of EG-LYS used (Fig. 1B). The growth inhibitory efficacies on E. faecalis (mean ± SD, 40.32 ± 24.27 Δlog_10_CFU/h/mg) were higher than those against the other tested bacterial strains, including *E. avium*, E. faecium, and GBS (−4.27 ± 3.47 Δlog_10_CFU/h/mg).

These results indicated that EG-LYS specifically inhibited the growth of E. faecalis by degrading its peptidoglycan.

### Construction of the false-negative model by coculturing GBS and E. faecalis.

**FIG 2:**
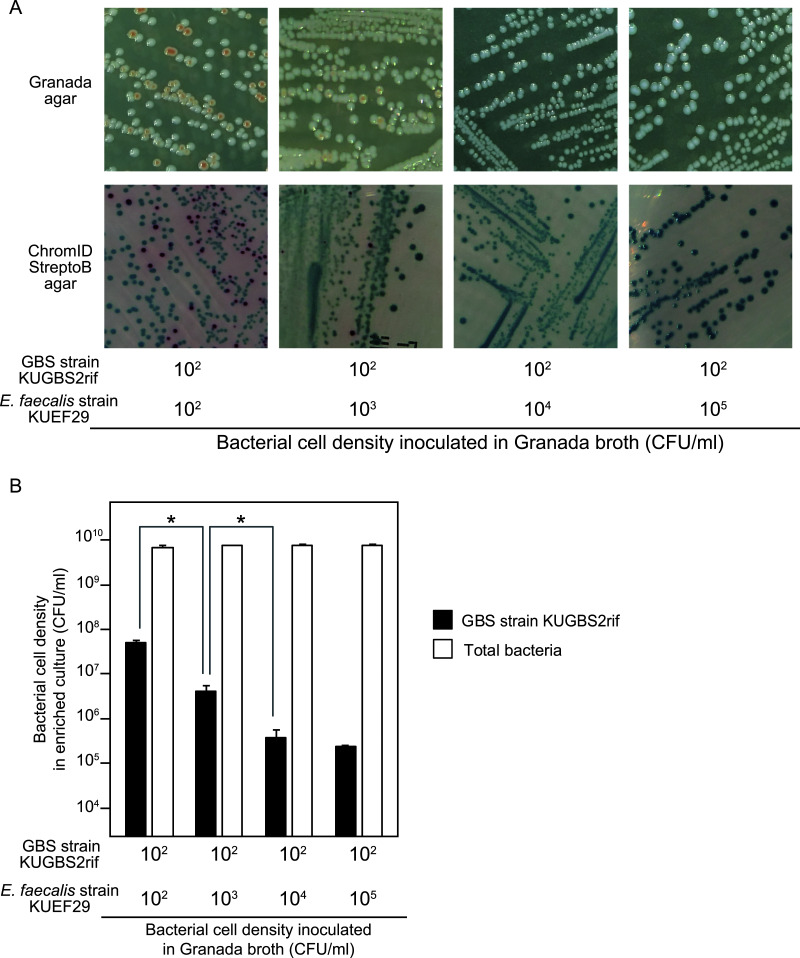
Construction of the false-negative model by coculturing GBS KUGBS2rif with Enterococcus faecalis KUEF29. GBS KUGBS2rif at 10^2^ CFU/ml was cocultured with E. faecalis KUEF29 at 10^2^, 10^3^, 10^4^, and 10^5^ CFU/ml in the Granada broth at 37°C for 24 h. The enriched culture was subcultured onto the Granada agar and the commercial chromogenic agar by stroke smear. (A) Colony appearance on Granada agar and commercial chromogenic agar. The GBS colony appeared orange on the Granada agar (top) and red on commercial chromogenic agar (bottom). GBS was detectable when KUGBS2rif at 10^2^ CFU/ml was inoculated with KUEF29 at 10^2^ and 10^3^ CFU/ml in Granada broth, whereas it was not detected with KUEF29 at ≥10^4^ CFU/ml. (B) Bacterial cell densities in the enriched cultures. The mean and SD of cell densities of KUGBS2rif (closed column) and total bacteria (open column) are presented with error bars (*n* = 3). Cell densities of total bacteria were measured to validate the experiment. According to one-way analysis of variance (ANOVA), no statistical difference was observed in the total bacteria (*P > *0.05), while it was observed in the KUGBS2rif (*P < *0.0001). According to the following Tukey’s *post hoc* test, the GBS densities decreased with statistical significance, as the inoculated densities of E. faecalis increased from 10^2^ and 10^3^ to 10^4^ CFU/ml. Statistical significance is denoted by an asterisk (*, *P < *0.001).

To test whether EG-LYS treatment suppresses E. faecalis growth and improves GBS detection, a false-negative model is essential. GBS strain KUGBS2rif at 10^2^ CFU/ml was cocultured with E. faecalis strain KUEF29 at 10^2^, 10^3^, 10^4^, or 10^5^ CFU/ml in Granada broth, and the culture broth was streaked on Granada and commercial chromogenic agars. KUGBS2rif was detected in the enriched broth inoculated with 10^2^ and 10^3^ CFU/ml KUEF29; however, it was not detected in enriched broth inoculated with 10^4^ and 10^5^ CFU/ml KUEF29 (Fig. 2A). GBS efficiently produces pigments under anaerobic conditions (23), and therefore the incubation was not the most suitable condition for GBS detection. However, GBS detection was comparable between the cultures on Granada agar and commercial chromogenic agar, based on colony color under these conditions. In addition, we analyzed the bacterial cell densities in these coculture samples after enrichment and found that KUGBS2rif densities were the lowest when KUEF29 inoculation densities ranged between 10^4^ and 10^5^ CFU/ml (Fig. 2B). KUGBS2rif densities increased gradually as KUEF29 inoculation density decreased to 10^3^ to 10^2^ CFU/ml. Thus, the coculture of single strains of KUEF29 at 10^4^ CFU/ml and KUGBS2rif at 10^2^ CFU/ml at inoculation was used as a false-negative model. In addition, the coculture of single strains of E. faecalis at 10^4^ CFU/ml and GBS at 10^2^ CFU/ml at inoculation was examined using 7 and 30 strains of GBS and E. faecalis, respectively. Upon testing 210 pairs of single strains of GBS and E. faecalis, GBS detection failed in all combinations (see Table S1 in the supplemental material). Therefore, the coculture of single strains of E. faecalis at 10^4^ CFU/ml and GBS at 10^2^ CFU/ml at inoculation was used as a false-negative model.

### Effect of EG-LYS treatment on GBS detection in the false-negative coculture model.

**FIG 3:**
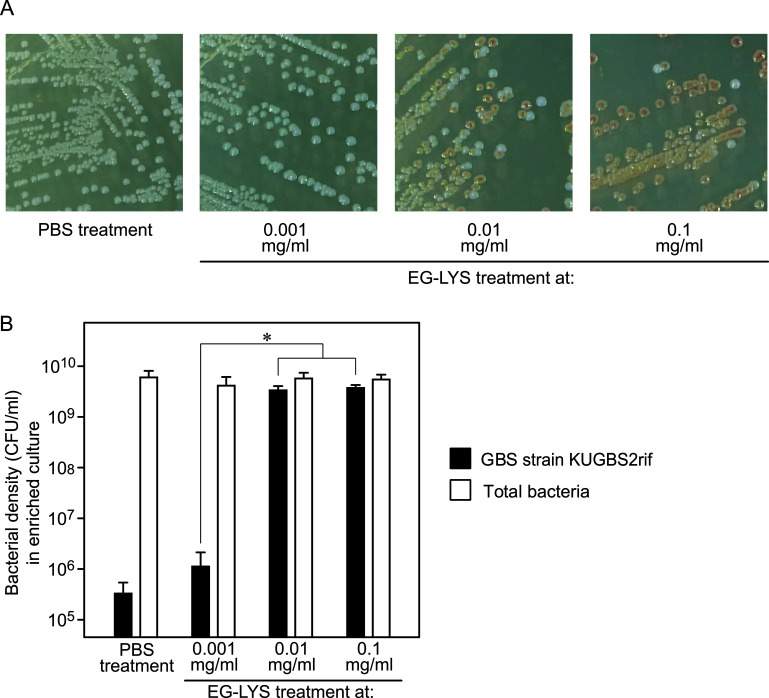
Effect of EG-LYS treatment in the false-negative model using GBS KUGBS2rif and E. faecalis KUEF29. (A) Appearance of bacterial colonies on Granada agar. On Granada agar, orange colonies are KUGBS2rif, while white colonies are KUEF29. Only E. faecalis appeared on the agar subcultured from the enriched culture with EG-LYS treatment at 0.001 mg/ml and with PBS treatment. Both KUGBS2rif and KUEF29 appeared on the Granada agar subcultured from the enriched cultures with EG-LYS treatments at 0.1 and 0.01 mg/ml. (B) Bacterial densities of KUGBS2rif and total bacteria in enrichment cultures with PBS and EG-LYS treatments. The false-negative model was treated with EG-LYS at 0.1, 0.01, and 0.001 mg/ml. As a negative control, the enriched culture with PBS treatment was used. The means and SD of bacterial densities are presented as column charts with error bars (*n* = 6). According to one-way ANOVA, no statistical difference was observed among the tested groups in the total bacteria (*P > *0.05), while it was observed in the KUGBS2rif strain (*P < *0.0001). According to the following Tukey’s *post hoc* test, the GBS densities increased with statistical significance, as the EG-LYS treatment increased from 0.001 mg/ml to 0.01 and 0.1 mg/ml. Statistical significance is denoted by an asterisk (*, *P < *0.001).

Different concentrations of EG-LYS (0.1, 0.01, and 0.001 mg/ml) were tested in the false-negative model with strains KUGBS2rif and KUEF29. We examined the appearance of GBS colonies on Granada agars after enrichment culture (Fig. 3A) and found that GBS colonies were detected from the samples treated with 0.1 and 0.01 mg/ml of EG-LYS, whereas they were undetectable from the culture treated with 0.001 mg/ml of EG-LYS and in the PBS-treated control. In addition, we measured the bacterial densities in the enrichment culture and found that the density of KUGBS2rif was approximately 10^9^ to 10^10^ CFU/ml in the EG-LYS treatment culture (0.1 and 0.01 mg/ml), whereas it was approximately 10^5^ to 10^6^ CFU/ml in the EG-LYS (0.001 mg/ml) and PBS treatment cultures (Fig. 3B). We also monitored the change in the bacterial densities over time; EG-LYS treatment at 0.1 and 0.01 mg/ml showed that the growth of KUEF29 was suppressed during the initial 3 h of incubation, possibly enabling the growth of GBS cells (see Fig. S2 in the supplemental material).

In the aforementioned 210 pairs of false-negative models, EG-LYS treatment at 0.1 and 0.01 mg/ml showed a GBS detection rate of 99.0% (208 out of 210 pairs) and 59.0% (114 out of 210 pairs), respectively (see Table S1 in the supplemental material). The reduction in EG-LYS concentration from 0.1 to 0.01 mg/ml approximately halved the E. faecalis-inhibitory effect. These results strongly indicate that EG-LYS treatment is useful in improving GBS detection. Considering these results, we used an EG-LYS concentration of 0.1 mg/ml in further experiments.

### Verification of EG-LYS treatment in prenatal GBS screening test using vaginal swabs.

Using 548 vaginal swabs collected at a clinical laboratory for unknown purposes, the effect of EG-LYS treatment on GBS detection was tested. The swabs were resuspended in a small volume of Granada broth, dispensed into a pair of Granada broths; one tube supplemented with EG-LYS at 0.1 mg/ml and the other with an equal volume of PBS. Tubes were incubated at 37°C for 24 h, and the enriched cultures were then streaked onto chromogenic agars, followed by colony identification using mass spectrometry. Bacterial colonies were observed in 371 samples, yet none were observed in the culture of the remaining 177 samples. Among the 371 samples, 98 and 86 samples were GBS-positive in EG-LYS and PBS treatment, respectively. Thus, the GBS detection ratio in EG-LYS-treated culture appeared to be 17.9% (98/548), whereas that in the PBS-treated culture was 15.7% (86/548 samples), consistent with the results of a culture-based prenatal GBS screening test reported in a previous study (24). GBS-positive samples from the EG-LYS-treated culture were consistently GBS positive in PBS-treated cultures, suggesting that the EG-LYS treatment did not interfere with the test results. Improved GBS detection ratios due to EG-LYS treatment appeared statistically significant (McNemar test, *P = *0.00151; Table 1), indicating that the EG-LYS treatment reduced the ratio of false-negative results without affecting specificity. False-negative samples accounted for approximately 2.2% (12 out of 548 samples) of the samples.

**TABLE 1:** Results of clinical examination

Detection of GBS	Treatment with EG-LYS in enrichment medium (% [no./total])	Treatment with PBS in enrichment medium (% [no./total])
Positive	17.9 (98/548)	15.7 (86/548)
Negative	82.1 (450/548)	84.3 (462/548)

In addition to the 12 false-negative samples described above, five GBS-positive samples showed increased number of GBS colonies on the chromogenic agar in the EG-LYS treatment compared to that in the PBS treatment (see Fig. S3 in the supplemental material). Among the 98 GBS-positive samples from the EG-LYS-treated enriched cultures, 17 samples, which included 12 false-negative samples and five GBS-positive samples with increased number of GBS colonies, were considered to improve the GBS detection. Thus, EG-LYS treatment contributed to an increase in GBS detection by 17.3% (i.e., 17/98 GBS-positive samples).

Note that this test has a few limitations. First, this test used vaginal swabs rather than vaginal-rectal swabs. The detection rate of GBS using vaginal-rectal swabs is higher than those using vaginal swabs, and therefore the use of vaginal-rectal swabs is recommended for better GBS recovery (4, 25). Second, fresh vaginal swab samples were not used. To accurately evaluate the effects of EG-LYS treatment in the clinical setting, the vaginal-rectal swab samples should be used in the culture-based prenatal GBS screening tests within 24 h. Third, the vaginal swabs were not collected for prenatal GBS screening test.

### Effect of EG-LYS treatment on the microbiota of enriched cultures.

**FIG 4:**
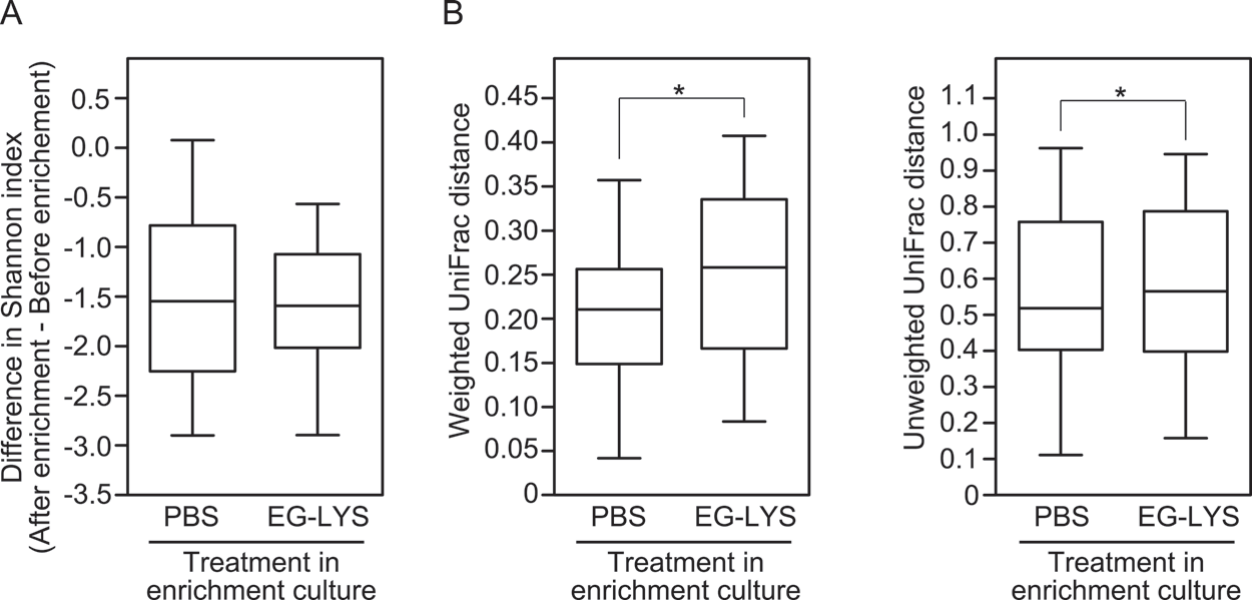
Analyses of microbial diversities of 17 pairs of swab-resuspended broth and enriched cultures treated with PBS and EG-LYS. (A) Differences in alpha diversity of Shannon index metric between before and after enrichments by PBS and EG-LYS treatments. No significant difference between EG-LYS and PBS treatments was observed. (B) Changes in microbial community structures following enrichment cultures between PBS and EG-LYS treatments. Weighted and unweighted UniFrac distances are shown on the left and right, respectively. Statistical analysis was conducted by Wilcoxon signed-rank test. Statistical significance is denoted by an asterisk (*, *P < *0.05).

The effects of EG-LYS treatment on the microbiota of enrichment culture were examined. The microbiota of the above-mentioned 17 samples were compared between the EG-LYS and PBS treatments. Seventeen pairs of one preculture sample and two postculture samples of EG-LYS and PBS treatments were analyzed using 16S rRNA amplicon sequencing. A total of 51 sequencing data (1,850,335 reads in total; 36,281 ± 6,578 reads/sequencing data) were obtained (see Fig. S4 in the supplemental material). Analysis of the microbial diversity indicated that there was no significant difference in the species richness between the PBS and EG-LYS treatments in the enrichment culture (Fig. 4); however, EG-LYS and PBS treatments caused phylogenetic changes in the bacterial community.

Moreover, the bacterial taxa that were significantly abundant in the enriched culture broths treated with EG-LYS and PBS were analyzed (Table 2). Bacterial taxa with <0.01% of relative abundance were classified together; 28 bacterial taxa at the species level were detected in the data set. Analysis of the data set using the Wilcoxon signed-rank test showed that only S. agalactiae and Enterococcus spp. were significantly more and less abundant, respectively, in the EG-LYS-treated enriched cultures than in the PBS-treated enriched cultures. EG-LYS treatment does not influence the population of other bacterial taxa in the clinical specimens. This result was mostly consistent with the results of the bacterial identification tests based on chromogenic agars followed by mass spectrometry (see Table S2 in the supplemental material). Thus, this microbiome analysis strongly corroborated the improvement in GBS detection using EG-LYS treatment without being influenced by other bacterial populations in the enrichment culture.

**TABLE 2:** Differential abundance analysis of microbiota in enriched cultures treated with EG-LYS and PBS

Bacterial taxonomy^*a*^	*P*	Adjusted *P*	Log_2_(fold change)	Ordering
Firmicutes, Bacilli, Lactobacillales, Streptococcaceae, Streptococcus agalactiae	0.00002	0.00043	−2.10	EG-LYS treatment → PBS treatment
*Firmicutes*, *Bacilli*, *Lactobacillales*, Enterococcaceae, Enterococcus (no species)	0.00348	0.04876	5.68	EG-LYS treatment ← PBS treatment
Other bacterial taxa	>0.05	>0.7		

a Bacterial taxonomy is shown in the order of phylum, class, order, family, and genus and species.

### Perspective of endolysin use in prenatal GBS screening tests.

Use of endolysin improved GBS detection by approximately 2.2% in this study. Approximately 900 thousand pregnancies were reported in Japan in 2018 (26). Assuming that GBS was detected in approximately 2.2% of pregnancies, using EG-LYS in prenatal GBS screening tests could reduce the chances of GBS infections in 19.8 thousand neonates at risk through prenatal and perinatal administration of antibiotics. Thus, EG-LYS treatment of the enrichment culture in GBS testing potentially reduces the risks of GBS infection in neonates from the relatively large population of GBS carriers by prenatal and perinatal prophylactic treatments of antibiotics. Moreover, the dissemination of drug-resistant GBS recently reported worldwide could be reduced by use of endolysin in the prenatal GBS screening test (27–29).

To introduce the use of endolysin as part of clinical tests, several hurdles must be overcome. (i) The storage stability of endolysin is important for stable supply. EG-LYS retained its potency for 6 months when stored in Granada broth at 4°C (see Fig. S5 in the supplemental material). (ii) Effective process development for endolysin can be achieved using the quality by design (QbD) approach for maintaining product quality (30). In the pharmaceutical industry, process development needs to be optimized by design of experiments (DoE) (31). (iii) We used liquid culture broth for enrichment; however, commercially available enrichment broths are typically semifluid. Application of endolysin to such typical forms of GBS enrichment broths should be optimized for user-friendliness. By overcoming the latter two hurdles, EG-LYS is expected to be applied in prenatal GBS screening tests in clinical settings.

While most endolysin products were effective against Gram-positive bacteria, those effective against Gram-negative bacteria have been recently reported (32). We hope to extend the use of endolysin to the clinical setting.

## MATERIALS AND METHODS

### Bacteria, culture media, and reagents.

We used 5, 30, 5, and 7 strains of *E. avium*, E. faecalis, E. faecium, and GBS, respectively, which were all isolated from clinical vaginal swabs and identified using mass spectrometry (17). In the case of GBS strains, the Lancefield B antigens were also tested using a kit (Strept LA “SEIKEN”; Denka Company, Niigata, Japan). In addition, we used E. faecalis ATCC 29212, Escherichia coli DH5α, E. coli NiCo21 (DE3) (New England BioLabs, Ipswich, MA), and a rifampin-resistant GBS KUGBS2rif strain derived from GBS KUGBS2 cultured in tryptic soy agar (TSA) supplemented with 20 μg/ml rifampin.

The enterococcal and streptococcal strains were cultured in tryptic soy broth (TSB) or TSA unless otherwise stated, while E. coli was cultured in Luria-Bertani media (LB medium [Miller]; Kanto Chemical Co., Tokyo, Japan).

Granada broth (25.0 g/liter proteose peptone no. 3, 14.0 g/liter soluble starch, 2.5 g/liter glucose, 1.0 g/liter pyruvic acid sodium salt, 0.1 g/liter cysteine hydrochloride, 0.3 g/liter magnesium sulfate, 11.0 g/liter 3-(*N*-morpholino)propane sulfonic acid, 10.7 g/liter disodium hydrogen phosphate, 0.5 mg/liter crystal violet, 10 mg/liter colistin sulfate, 10 mg/liter metronidazole, and 15 mg/liter nalidixic acid [pH 7.4]) was prepared as described previously with a slight modification (17).

TSA and rifampin-supplemented TSA were used to score the total cell count and the count for strain KUGBS2rif, respectively.

Granada agar, which was prepared by supplementing Granada broth with 1.5% (wt/vol) agar, and/or ChromID StreptoB agars (Sysmex bioMérieux Co., Ltd., Tokyo, Japan) was used to detect GBS colonies, based on colony pigments.

Culture media were purchased from Beckton Dickinson and Co. (Franklin Lakes, NJ) unless otherwise stated. Other reagents were purchased from Nacalai Tesque (Kyoto, Japan) or Fujifilm Wako Pure Chemicals (Osaka, Japan) unless otherwise stated. All bacteria were cultured aerobically at 37°C unless otherwise stated.

### Phage isolation and genome sequencing.

Enterococcal phages were isolated from sewage influent water collected in Tokyo, Japan, using E. faecalis strain KUEF2 as an indicator, by a method described elsewhere (17). Phages were purified after large-scale phage amplification, and phage genomic DNA was isolated using methods described previously (17, 33). A shotgun library was prepared for each phage DNA using the GS FLX Titanium rapid library preparation kit (Roche Diagnostics, Indianapolis, IN) according to the manufacturer’s instructions. The libraries were analyzed using a GS Junior 454 sequencer (Roche Diagnostics). The sequence reads were assembled using Newbler (version 3.0; 454 Life Sciences, Branford, CT) (34). The draft genome sequence obtained through *de novo* assembly of 5,153 shotgun pyrosequencing reads, 24.0 of the peak depth, was 56,582 bp.

The genome sequence was analyzed using the BLASTn tool at National Center for Biotechnology Information (NCBI; https://blast.ncbi.nlm.nih.gov/Blast.cgi; accessed 20 January 2020). The genome was annotated using the prokaryotic genome annotation pipeline DFAST (https://dfast.nig.ac.jp/; accessed 20 February 2019) (35). The predicted gene products were analyzed using the BLASTp tool at NCBI (https://blast.ncbi.nlm.nih.gov/Blast.cgi; accessed 20 February 2019).

### Cloning and protein expression.

The phage phiM1EF2 sequence was amplified by PCR (HiFi DNA polymerase; Kapa Biosystems, Wilmington, MA) using the primer set phiM1EF2LYS_F (5′-CGGTACCCGGGGATC-GGTACC-ATGGTTAAAGTAAACGATGTACTAAGCTATG-3′) and phiM1EF2LYS_R2 (5′-CGACTCTAGAGGATC-AAGCTT-TTAATGATGATGATGATGATG-TACTTTAACTTGTGGTAAAGCTGCTTCC-3′), with phage phiM1EF2 genomic DNA as the template. The PCR product was cloned into pUC19 using an In-Fusion HD cloning kit (TaKaRa Bio, Shiga, Japan). The fragment cloned accurately in pUC19 was excised with HindIII and KpnI and subcloned into the expression vector pCold III (TaKaRa Bio). This plasmid for the recombinant ORF60 expression was designated pCold III *orf60*. The plasmids were used to transform E. coli strains DH5α and NiCo21 (DE3) for cloning and protein expression, respectively.

To overexpress the recombinant protein, E. coli NiCo21 (DE3) harboring the pCold III *orf60* plasmid was exponentially grown to an optical density at 600 nm (OD_600_) of 0.2 to 0.3, in 250 ml LB medium and then allowed to stand for 30 min on ice. The culture was supplemented with 1 mM isopropyl-β-d-thiogalactopyranoside, and the bacteria were cultured aerobically for 36 h at 15°C. The culture was centrifuged (10,000 × *g*, 5 min, 4°C), the cell pellets were resuspended in 25 ml of lysis buffer (100 mM sodium phosphate and 300 mM NaCl [pH 7.8]), and sonicated (5-s pulses at 5-s intervals 60 times) on ice, using a Q700 sonicator (Qsonica, Newtown, CT). The cell lysate was centrifuged (8,000 × *g*, 10 min, 4°C), and the supernatant was incubated with gentle mixing overnight at 4°C with 250 μl Co^2+^-agarose resin (ProteNova, Kagawa, Japan). The resin was washed with lysis buffer and then packed into an open column. The column was eluted sequentially using lysis buffer supplemented with 5 mM, 50 mM, 350 mM, and 500 mM imidazole. The eluates of 350 mM and 500 mM imidazole buffer were enriched with the recombinant proteins. The elutes were dialyzed overnight at 4°C against PBS (pH 7.4).

Protein concentration was measured using Bradford reagent (TaKaRa Bradford Protein assay kit; TaKaRa Bio), with bovine serum albumin as the standard.

### Analysis of peptidoglycan hydrolysis activity.

Peptidoglycan suspensions from E. faecalis, E. faecium, E. avium, and GBS were prepared as follows. An exponential-phase culture at an OD_600_ of 0.6 was suspended in 4% sodium dodecyl sulfate (SDS), autoclaved (15 min, 121°C), and washed six times with deionized water. Crude peptidoglycan was suspended in PBS. Bacterial cells to be used for endolysin substrate were grown until the mid-log phase, washed with PBS once, and suspended in PBS.

The initial turbidity of suspensions (peptidoglycan or intact cells in 100 μl of PBS) in an uncoated sterile polystyrene 96-well plate were adjusted to an optical density of 595 nm (OD_595_) of 0.2 to 0.3, measured using a Multiskan JX spectrophotometer (Thermo Labsystems, Stockholm, Sweden). Serially diluted purified recombinant protein (50 μl) was added to the well at 0.1 and 0.01 mg protein/ml. PBS was used as a negative control. Plates were incubated at 37°C with stirring. Turbidity of the crude peptidoglycan or the cells suspended in PBS was measured at 0, 5, 15, 30, and 60 min. The mean value of three wells was used as the representative data for each treatment. The reaction velocity was measured in ΔOD_595_/min/mg, which is defined as the turbidity reduction rate per time and the amount of protein used, as described in Fig. S6 in the supplemental material.

### Analysis of the growth inhibitory efficiency against a single bacterial species.

The overnight bacterial culture in TSB was resuspended in double strength TSB (2×TSB) at 2.0 to 3.0 × 10^4^ CFU/ml. An equal volume of counterpart bacterial suspension in 2×TSB was mixed with PBS (negative control) or the purified protein solution (0.02 mg/ml). After incubation for 3 to 6 h, the viable numbers on TSA were counted. The growth-inhibitory efficiency against each bacterium in Δlog_10_CFU/h/mg was calculated from the difference in bacterial growth between EG-LYS and PBS treatments divided by the incubation time and amount of protein used, as described in Fig. S7 in the supplemental material. The experiments were repeated at least three times, and the mean value was obtained.

### Setting of the false-negative model by coculture of E. faecalis and GBS.

Bacteria were grown until the mid-log phase (OD_600_ = 0.3 to 0.4) and diluted to the desired densities in Granada broth. The GBS strain KUGBS2rif at 2.0 × 10^2^ to 4.0 × 10^2^ CFU/ml was cocultured with E. faecalis strain KUEF29 at 2.0 × 10^2^ to 4.0 × 10^2^ CFU/ml, 2.0 × 10^3^ to 4.0 × 10^3^ CFU/ml, 2.0 × 10^4^ to 4.0 × 10^4^ CFU/ml, or 2.0 × 10^5^ to 4.0 × 10^5^ CFU/ml. The enrichment culture was performed for 24 h in air with 5% CO_2_. The bacterial densities of KUGBS2rif and total bacteria in the enriched cultures were measured using appropriate agar media, as described above. The enriched culture was then streaked onto the Granada agar and ChromID StreptoB agars. Following the 24 h of incubation, the growth of GBS colonies on the agars was visually evaluated based on the color. Experiments were repeated three times.

We cocultured 210 pairs of single strains of bacteria, using 7 strains of GBS and 30 strains of E. faecalis, at the inoculation bacterial density of 10^2^ CFU/ml GBS and 10^4^ CFU/ml E. faecalis, for 24 h in air with 5% CO_2_. The enriched cultures were streaked onto Granada agar and then incubated for 24 to 48 h as described above. GBS detection was performed visually based on the color of GBS colonies. Experiments were repeated three times.

### Determining effectiveness of purified protein in the false-negative model.

The purified proteins at desired concentrations were added to the false-negative models containing 10^2^ and 10^4^ CFU/ml of GBS and E. faecalis, respectively, in Granada broth. As a negative control, the purified protein was replaced with PBS. The culture broth was incubated for 24 h in air with 5% CO_2_. In the false-negative model using the strains KUGBS2rif and KUEF29, the bacterial densities in the enriched cultures were measured. The enriched cultures were subcultured onto Granada agar by stroke smearing. After incubation in 5% CO_2_ for 24 to 48 h, the appearance of colonies was visually observed, and the GBS detection was performed. Experiments were repeated at least thrice.

### Examining the effectiveness of purified protein in clinical specimens.

Residual vaginal swabs with anonymized personal and clinical information were used in this study. The vaginal swab samples collected from patients at the local clinical laboratory and subjected to clinical laboratory tests were used in this study. The results of the clinical laboratory tests were not provided in this study for protection of privacy. The swabs stored at 4°C within 1 to 2 weeks following the sample collection were used.

The swabs were resuspended in 2.5 ml of Granada broth and dispensed into two 0.9-ml aliquots for the enrichment cultures. Remaining broth (approximately 0.6 ml) was stored at −80°C. Before enrichment culture, 0.9 ml of the swab-resuspended Granada broth was supplemented with 0.1 ml of PBS or purified protein solution and then incubated for 24 h. The enriched culture was streaked onto ChromID StreptoB agars and incubated for 24 to 48 h. The bacterial colonies were isolated and subcultured on 5% sheep blood agar plates (Eiken Chemical Co., Ltd., Tokyo, Japan). The isolated bacteria were identified using a microflex system (Bruker Daltonics, Bremen, Germany). The protein profile of each bacterium was analyzed using the matrix-assisted laser desorption ionization (MALDI) Biotyper v3.1 with the most updated spectrum library, v9 (8,468 spectra). All cultures were incubated at 35°C under aerobic conditions.

The experiment was approved by the Kitasato Institute Hospital Research Ethics Committee (approval no. 19060). The inoculated Granada broth and enriched culture broths were stored at −80°C for microbiota analysis.

### 16S rRNA amplicon sequencing and analysis.

DNA was extracted from the swab-resuspended broths and the enriched culture broths, using an Isospin fecal DNA kit (Nippon Gene, Tokyo, Japan). The V3-V4 regions of the 16S rRNA gene were amplified by PCR and analyzed using a MiSeq instrument with a MiSeq reagent kit v3 (600 cycles; Illumina) as described previously (36). The sequence data were processed using Quantitative Insights Into Microbial Ecology 2 (QIIME 2) v2020.11.0 (37). The DADA2 software package v2020.8.0 incorporated in QIIME 2 was used to correct sequence errors and construct an amplicon sequence variant table. Microbial taxonomy was assigned using a naive Bayes classifier trained on the SILVA 138 99% database (38). Statistical analysis was performed using R implemented in QIIME 2. The differential abundance analysis was performed using DAtest software v2.7.12, installed in R v4.0.3 (39, 40).

### Statistical analysis.

Statistical analyses were performed using Prism 4 (GraphPad Software, La Jolla, CA), or R version 4.0.3 (41). A *P* value of <0.05 was considered statistically significant.

### Data availability.

 The draft genome sequence of phage phiM1EF2 was deposited in GenBank under accession number LC547238. The sequencing data of the 16S rRNA gene (V3-V4 region) amplicons were deposited under BioProject no. PRJDB10939.

## References

[1] Hanna M, Noor A. 2020. *Streptococcus* group B. StatPearls Publishing, Treasure Island, FL. 31985936

[2] Raabe VN, Shane AL. 2019. Group B *Streptococcus* (*Streptococcus agalactiae*). Microbiol Spectr 7. doi:10.1128/microbiolspec.GPP3-0007-2018. PMC6432937 30900541

[3] Cagno CK, Pettit JM, Weiss BD. 2012. Prevention of perinatal group B streptococcal disease: updated CDC guideline. Am Fam Physician 86:59–65. 22962913

[4] Filkins L, Hauser JR, Robinson-Dunn B, Tibbetts R, Boyanton BL, Revell P. 2020. American Society for Microbiology provides 2020 guidelines for detection and identification of group B *Streptococcus*. J Clin Microbiol 59:e01230-20. doi:10.1128/JCM.01230-20. 33115849 PMC7771461

[5] de la Rosa M, Perez M, Carazo C, Pareja L, Peis JI, Hernandez F. 1992. New Granada medium for detection and identification of group B streptococci. J Clin Microbiol 30:1019–1021. doi:10.1128/jcm.30.4.1019-1021.1992. 1572958 PMC265207

[6] Carvalho MDG, Facklam R, Jackson D, Beall B, McGee L. 2009. Evaluation of three commercial broth media for pigment detection and identification of a group B *Streptococcus* (*Streptococcus agalactiae*). J Clin Microbiol 47:4161–4163. doi:10.1128/JCM.01374-09. 19812277 PMC2786674

[7] Dunne WM, Jr, Holland-Staley CA. 1998. Comparison of NNA agar culture and selective broth culture for detection of group B streptococcal colonization in women. J Clin Microbiol 36:2298–2300. doi:10.1128/JCM.36.8.2298-2300.1998. 9666009 PMC105035

[8] Park CJ, Vandel NM, Ruprai DK, Martin EA, Gates KM, Coker D. 2001. Detection of group B streptococcal colonization in pregnant women using direct latex agglutination testing of selective broth. J Clin Microbiol 39:408–409. doi:10.1128/JCM.39.1.408-409.2001. 11191227 PMC87747

[9] Binghuai L, Yanli S, Shuchen Z, Fengxia Z, Dong L, Yanchao C. 2014. Use of MALDI-TOF mass spectrometry for rapid identification of group B *Streptococcus* on chromID Strepto B agar. Int J Infect Dis 27:44–48. doi:10.1016/j.ijid.2014.06.023. 25220051

[10] Jordan JA, Hall G, Davis T. 2010. Multicenter study evaluating performance of the Smart Group B *Streptococcus* (GBS) assay using an enrichment protocol for detecting GBS colonization in patients in the antepartum period. J Clin Microbiol 48:3193–3197. doi:10.1128/JCM.00106-10. 20668132 PMC2937680

[11] Shin JH, Pride DT. 2019. Comparison of three nucleic acid amplification tests and culture for detection of group B *Streptococcus* from enrichment broth. J Clin Microbiol 57:e01958-18. doi:10.1128/JCM.01958-18. 30944190 PMC6535594

[12] Ross A, Ward S, Hyman P. 2016. More is better: selecting for broad host range bacteriophages. Front Microbiol 7:1352. doi:10.3389/fmicb.2016.01352. 27660623 PMC5014875

[13] Schmelcher M, Donovan DM, Loessner MJ. 2012. Bacteriophage endolysins as novel antimicrobials. Future Microbiol 7:1147–1171. doi:10.2217/fmb.12.97. 23030422 PMC3563964

[14] Pohane AA, Jain V. 2015. Insights into the regulation of bacteriophage endolysin: multiple means to the same end. Microbiology (Reading) 161:2269–2276. doi:10.1099/mic.0.000190. 26419630

[15] Azam AH, Tanji Y. 2019. Bacteriophage-host arm race: an update on the mechanism of phage resistance in bacteria and revenge of the phage with the perspective for phage therapy. Appl Microbiol Biotechnol 103:2121–2131. doi:10.1007/s00253-019-09629-x. 30680434

[16] Shemyakin IG, Firstova VV, Fursova NK, Abaev IV, Filippovich SY, Ignatov SG, Dyatlov IA. 2020. Next-generation antibiotics, bacteriophage endolysins, and nanomaterials for combating pathogens. Biochemistry (Mosc) 85:1374–1388. doi:10.1134/S0006297920110085. 33280580

[17] Uchiyama J, Matsui H, Murakami H, Kato SI, Watanabe N, Nasukawa T, Mizukami K, Ogata M, Sakaguchi M, Matsuzaki S, Hanaki H. 2018. Potential application of bacteriophages in enrichment culture for improved prenatal *Streptococcus agalactiae* screening. Viruses 10:552. doi:10.3390/v10100552. 30308933 PMC6213948

[18] Oechslin F. 2018. Resistance development to bacteriophages occurring during bacteriophage therapy. Viruses 10:351. doi:10.3390/v10070351. 29966329 PMC6070868

[19] Jault P, Leclerc T, Jennes S, Pirnay JP, Que YA, Resch G, Rousseau AF, Ravat F, Carsin H, Le Floch R, Schaal JV, Soler C, Fevre C, Arnaud I, Bretaudeau L, Gabard J. 2019. Efficacy and tolerability of a cocktail of bacteriophages to treat burn wounds infected by *Pseudomonas aeruginosa* (PhagoBurn): a randomised, controlled, double-blind phase 1/2 trial. Lancet Infect Dis 19:35–45. doi:10.1016/S1473-3099(18)30482-1. 30292481

[20] Grieco SH, Wong AY, Dunbar WS, MacGillivray RT, Curtis SB. 2012. Optimization of fermentation parameters in phage production using response surface methodology. J Ind Microbiol Biotechnol 39:1515–1522. doi:10.1007/s10295-012-1148-3. 22714954

[21] Maciejewska B, Olszak T, Drulis-Kawa Z. 2018. Applications of bacteriophages versus phage enzymes to combat and cure bacterial infections: an ambitious and also a realistic application? Appl Microbiol Biotechnol 102:2563–2581. doi:10.1007/s00253-018-8811-1. 29442169 PMC5847195

[22] Mondal SI, Draper LA, Ross RP, Hill C. 2020. Bacteriophage endolysins as a potential weapon to combat *Clostridioides difficile* infection. Gut Microbes 12:1813533. doi:10.1080/19490976.2020.1813533. 32985336 PMC7524323

[23] Rosa-Fraile M, Rodriguez-Granger J, Cueto-Lopez M, Sampedro A, Gaye EB, Haro JM, Andreu A. 1999. Use of Granada medium to detect group B streptococcal colonization in pregnant women. J Clin Microbiol 37:2674–2677. doi:10.1128/JCM.37.8.2674-2677.1999. 10405420 PMC85311

[24] Carrillo-Avila JA, Gutierrez-Fernandez J, Gonzalez-Espin AI, Garcia-Trivino E, Gimenez-Lirola LG. 2018. Comparison of qPCR and culture methods for group B *Streptococcus* colonization detection in pregnant women: evaluation of a new qPCR assay. BMC Infect Dis 18:305. doi:10.1186/s12879-018-3208-4. 29976153 PMC6034337

[25] El Aila NA, Tency I, Claeys G, Saerens B, Cools P, Verstraelen H, Temmerman M, Verhelst R, Vaneechoutte M. 2010. Comparison of different sampling techniques and of different culture methods for detection of group B streptococcus carriage in pregnant women. BMC Infect Dis 10:285. doi:10.1186/1471-2334-10-285. 20920213 PMC2956727

[26] Director-General for Statistics, Information Policy and Policy Evaluation. 2018. Vital statistics of Japan 2018. Ministry of Health, Labour, and Welfare, Japan.

[27] Plainvert C, Hays C, Touak G, Joubrel-Guyot C, Dmytruk N, Frigo A, Poyart C, Tazi A. 2020. Multidrug-resistant hypervirulent group B *Streptococcus* in neonatal invasive infections, France, 2007–2019. Emerg Infect Dis 26:2721–2724. doi:10.3201/eid2611.201669. 33079049 PMC7588536

[28] Gao K, Guan X, Zeng L, Qian J, Zhu S, Deng Q, Zhong H, Pang S, Gao F, Wang J, Long Y, Chang CY, Liu H. 2018. An increasing trend of neonatal invasive multidrug-resistant group B *Streptococcus* infections in southern China, 2011–2017. Infect Drug Resist 11:2561–2569. doi:10.2147/IDR.S178717. 30573985 PMC6292236

[29] Kimura K, Nagano N, Nagano Y, Suzuki S, Wachino J, Shibayama K, Arakawa Y. 2013. High frequency of fluoroquinolone- and macrolide-resistant streptococci among clinically isolated group B streptococci with reduced penicillin susceptibility. J Antimicrob Chemother 68:539–542. doi:10.1093/jac/dks423. 23111853

[30] Rathore AS. 2009. Roadmap for implementation of quality by design (QbD) for biotechnology products. Trends Biotechnol 27:546–553. doi:10.1016/j.tibtech.2009.06.006. 19647883

[31] Politis SN, Colombo P, Colombo G, Dimitrios DM. 2017. Design of experiments (DoE) in pharmaceutical development. Drug Dev Ind Pharm 43:889–901. doi:10.1080/03639045.2017.1291672. 28166428

[32] Lai WCB, Chen X, Ho MKY, Xia J, Leung SSY. 2020. Bacteriophage-derived endolysins to target Gram-negative bacteria. Int J Pharm 589:119833. doi:10.1016/j.ijpharm.2020.119833. 32877733

[33] Nasukawa T, Uchiyama J, Taharaguchi S, Ota S, Ujihara T, Matsuzaki S, Murakami H, Mizukami K, Sakaguchi M. 2017. Virus purification by CsCl density gradient using general centrifugation. Arch Virol 162:3523–3528. doi:10.1007/s00705-017-3513-z. 28785814

[34] Silva GG, Dutilh BE, Matthews TD, Elkins K, Schmieder R, Dinsdale EA, Edwards RA. 2013. Combining *de novo* and reference-guided assembly with scaffold_builder. Source Code Biol Med 8:23. doi:10.1186/1751-0473-8-23. 24267787 PMC4177539

[35] Tanizawa Y, Fujisawa T, Nakamura Y. 2018. DFAST: a flexible prokaryotic genome annotation pipeline for faster genome publication. Bioinformatics 34:1037–1039. doi:10.1093/bioinformatics/btx713. 29106469 PMC5860143

[36] Uchiyama J, Murakami H, Sato R, Mizukami K, Suzuki T, Shima A, Ishihara G, Sogawa K, Sakaguchi M. 2020. Examination of the fecal microbiota in dairy cows infected with bovine leukemia virus. Vet Microbiol 240:108547. doi:10.1016/j.vetmic.2019.108547. 31902503

[37] Bolyen E, Rideout JR, Dillon MR, Bokulich NA, Abnet CC, Al-Ghalith GA, Alexander H, Alm EJ, Arumugam M, Asnicar F, Bai Y, Bisanz JE, Bittinger K, Brejnrod A, Brislawn CJ, Brown CT, Callahan BJ, Caraballo-Rodríguez AM, Chase J, Cope EK, Da Silva R, Diener C, Dorrestein PC, Douglas GM, Durall DM, Duvallet C, Edwardson CF, Ernst M, Estaki M, Fouquier J, Gauglitz JM, Gibbons SM, Gibson DL, Gonzalez A, Gorlick K, Guo J, Hillmann B, Holmes S, Holste H, Huttenhower C, Huttley GA, Janssen S, Jarmusch AK, Jiang L, Kaehler BD, Kang KB, Keefe CR, Keim P, Kelley ST, Knights D, et al. 2019. Reproducible, interactive, scalable and extensible microbiome data science using QIIME 2. Nat Biotechnol 37:852–857. doi:10.1038/s41587-019-0209-9. 31341288 PMC7015180

[38] Quast C, Pruesse E, Yilmaz P, Gerken J, Schweer T, Yarza P, Peplies J, Glockner FO. 2013. The SILVA ribosomal RNA gene database project: improved data processing and web-based tools. Nucleic Acids Res 41:D590–D596. doi:10.1093/nar/gks1219. 23193283 PMC3531112

[39] Russel J, Thorsen J, Brejnrod AD, Bisgaard H, Sørensen SJ, Burmølle M. 2018. DAtest: a framework for choosing differential abundance or expression method. biorXiv. doi:10.1101/241802v1.

[40] Morton JT, Marotz C, Washburne A, Silverman J, Zaramela LS, Edlund A, Zengler K, Knight R. 2019. Establishing microbial composition measurement standards with reference frames. Nat Commun 10:2719. doi:10.1038/s41467-019-10656-5. 31222023 PMC6586903

[41] R Core Team. 2018. R: a language and environment for statistical computing. R Foundation for Statistical Computing, Vienna, Austria.

