# Supplementary material for: Use of Recombinant Endolysin to Improve Accuracy of Group B Streptococcus Tests
Source: Microbiol Spectr. 2021 Aug 11;9(1):10.1128/spectrum.00077-21. doi: 10.1128/spectrum.00077-21 (PMC8552716; doi:10.1128/spectrum.00077-21)
Supplement: SUPPLEMENTAL FILE 1 — Supplemental material. Download SPECTRUM00077-21_Supp_1_seq10.pdf, PDF file, 1.0 MB [file spectrum00077-21_supp_1_seq10.pdf]

**Supplementary Table S1.** Results of PBS and EG-LYS treatments on the false-negative model, using 210 pairs of single *E. faecalis* and GBS strains.

| GBS detection[a] | Untreatment/ PBS treatment | EG-LYS treatment at[b]: | |
|---|---|---|---|
| | | 0.01 mg/mL | 0.1 mg/mL |
| Detected | 0% (0/210) | 59.0% (124/210) | 99.0% (208/210) |
| Not detected | 100% (210/210) | 41.0% (86/210) | 1.0% (2/210) |

[a], The result of PBS treatment was statistically different from those of EG-LYS treatments at 0.01 mg/mL and 0.1 mg/mL ($P < 0.001$; McNemar's test).

[b], The result of EG-LYS treatment at 0.1 mg/mL was statistically different from that at 0.01 mg/mL ($P < 0.001$; McNemar's test).

**Supplementary Table S2.** Identification of bacterial colonies on chromogenic agars subcultured from seventeen paired enriched culture broths treated with PBS and EG-LYS.

| Sample No.[a] | Identified bacteria | |
|---|---|---|
| | **PBS treatment[b]** | **EG-LYS treatment** |
| 1 | *Enterococcus faecalis* | *Streptococcus agalactiae*<br>*Enterococcus faecalis* |
| 2 | *Enterococcus faecalis* | *Streptococcus agalactiae* |
| 3 | *Enterococcus faecalis* | *Streptococcus agalactiae*<br>*Enterococcus faecalis* |
| 4 | *Enterococcus faecalis* | *Streptococcus agalactiae*<br>*Enterococcus faecalis* |
| 5 | *Enterococcus casseliflavus* | *Streptococcus agalactiae* |
| 6 | *Enterococcus faecalis* | *Streptococcus agalactiae* |
| 7 | Not detected | *Streptococcus agalactiae* |
| 8 | Not detected | *Streptococcus agalactiae* |
| 9 | *Streptococcus gallolyticus* | *Streptococcus agalactiae* |
| 10 | Not detected | *Streptococcus agalactiae* |
| 11 | *Enterococcus faecalis* | *Streptococcus agalactiae* |
| 12 | *Enterococcus faecalis* | *Streptococcus agalactiae* |
| 13 | *Streptococcus agalactiae*<br>*Enterococcus faecalis*<br>*Candida albicans* | *Streptococcus agalactiae*<br>*Enterococcus faecalis* |
| 14 | *Streptococcus agalactiae* | *Streptococcus agalactiae* |
| 15 | *Streptococcus agalactiae*<br>*Enterococcus faecalis* | *Streptococcus agalactiae*<br>*Enterococcus gallinarum* |
| 16 | *Streptococcus agalactiae* | *Streptococcus agalactiae* |
| 17 | *Streptococcus agalactiae* | *Streptococcus agalactiae* |

[a], The sample Nos. 1 to 12 are false-negative sample; the sample Nos. 13 to 17 are GBS-positive samples with increased GBS detectability by EG-LYS treatment.
[b], No colonies were observed on the chromogenic agars treated with PBS, which are designated as "Not detected".

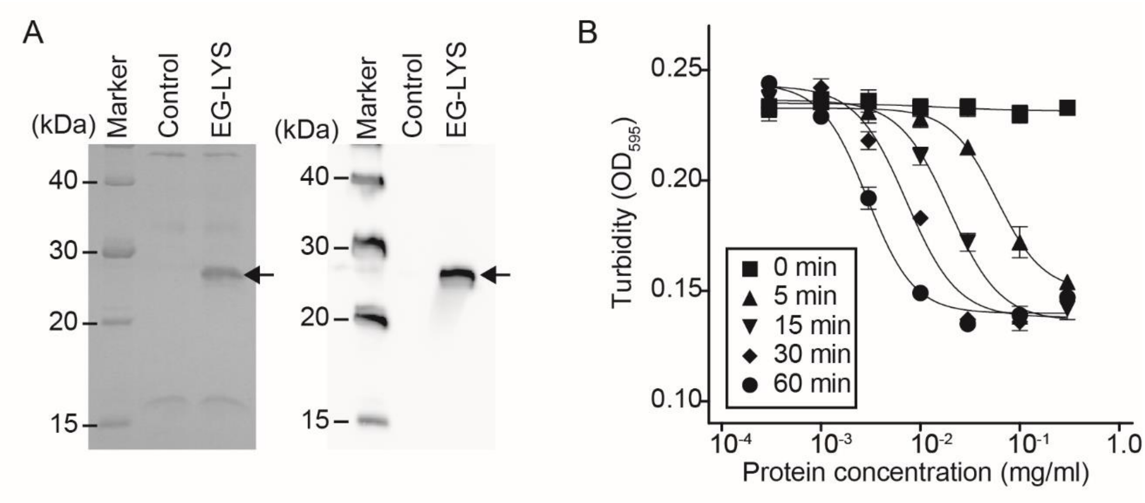


**Supplementary Fig. S1.** Characteristics of EG-LYS. (A) SDS-PAGE and western blot analyses. The corresponding elute from *E. coli* NiCo21 (DE3) [pCold III] to the EG-LYS elutes is designated as "control", which was used as a negative control. On the leftmost lanes, a molecular weight standard (XL-Ladder Broad, APRO Science) with molecular weights is shown. The protein samples were separated in 12.5% SDS-PAGE gel. An arrow indicates the recombinant ORF60. In the SDS-PAGE analysis (left), the EG-LYS (1.4 μg) and an equal volume of control were stained with Coomassie Brilliant Blue R-250. According to the analysis of band thicknesses in the SDS-PAGE image using Image J version 1.52, EG-LYS was calculated to have an average recombinant ORF60 content of 57.1% (SD, 1.0%; n = 3). Moreover, the proteins separated by SDS-PAGE were transferred to a polyvinylidene difluoride (PVDF) membrane (Amersham Hybond; 0.2 μm PVDF; GE Healthcare, Chicago, IL, USA). The blotted membrane was blocked with 3% skimmed milk in PBS with 0.05% Tween 20 (PBS-T) by incubating at 4 °C overnight, then washing with PBS-T (3 times for 5 min). The membrane was incubated with anti-6× His-tag mouse antibody conjugated with horseradish peroxidase (Anti-His-tag mAb-HRP-Direct; Medical and Biological Laboratories, Nagoya, Japan), diluted 1∶5,000 with 1% skimmed milk in PBS-T, for 1 h at room temperature (i.e., 20–25 °C). The membranes were washed (3 times for 5 min) in PBS-T. Immunoblot signals were detected using an ECL start western blotting detection system (GE Healthcare), and visualized using Image Quant LAS 4000 mini (GE Healthcare). In the western blot analysis (right), EG-LYS (0.17 μg) and an equal volume of control were analyzed. (B) Dose-dependent activity of EG-LYS for *E. faecalis* peptidoglycan. Peptidoglycan was treated with EG-LYS at 0.3, 0.1, 0.03, 0.01, 0.003, and 0.001 mg/ml. The turbidity was measured at 0, 5, 15, 30, and 60 min. The dose-dependency was analyzed from the data obtained through

the experiments repeated six times. In the square, each shape represents the treatment time of EG-LYS. Sigmoidal curves were fitted to the graph using GraphPad Prism 4 software (GraphPad Software, La Jolla, CA).

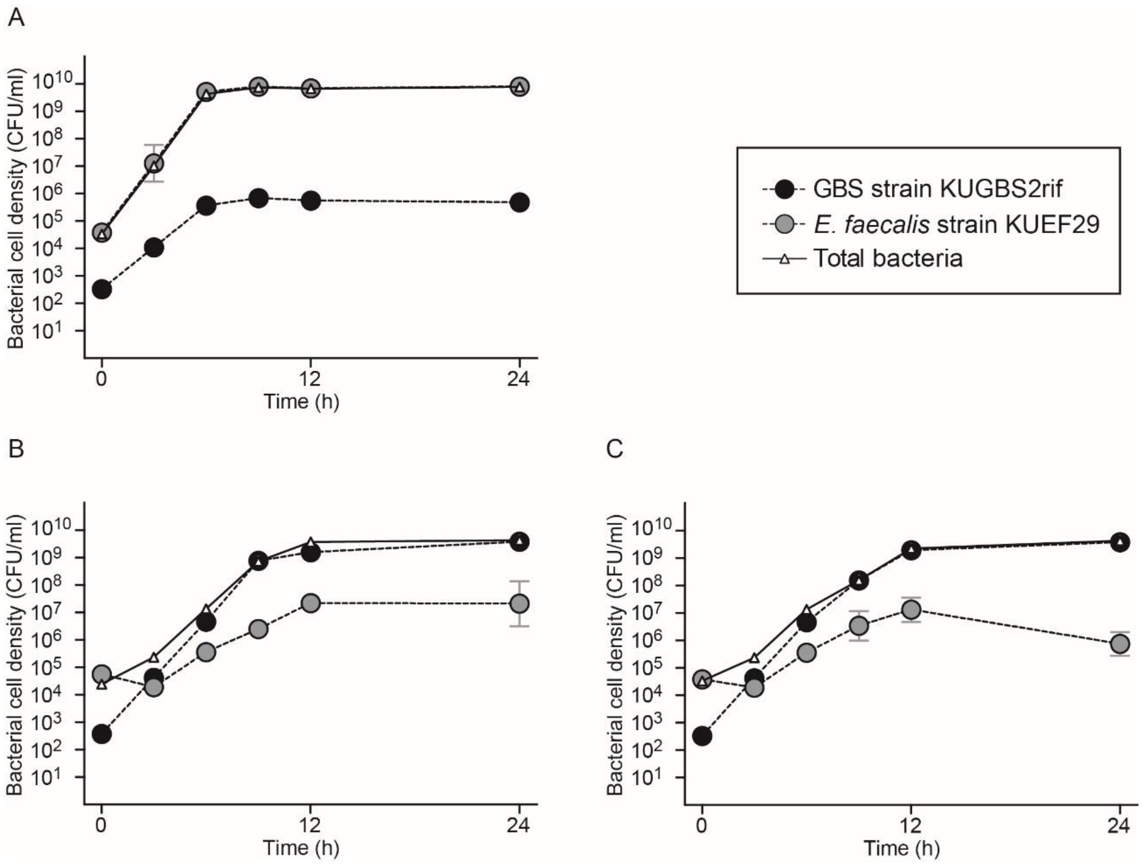


**Supplementary Fig. S2.** Change in bacterial cell densities over time in the KUGBS2rif-and-KUEF29 false-negative model treated with PBS and EG-LYS. The cell densities of GBS strain KUGBS2rif, *E. faecalis* strain KUEF29, and total bacteria in the enrichment culture were measured on TSA containing 20 µg/ml rifampicin, *Enterococcus*-selective agar (EF Agar Base "Nissui"; Nissui Pharmaceutical Co., Tokyo, Japan), and TSA, respectively. (A) PBS treatment. (B) EG-LYS treatment at 0.01 mg/ml, and (C) 0.1 mg/ml. The means with SDs are shown as symbols with error bars (n = 3). In the top right square, the symbols are described.

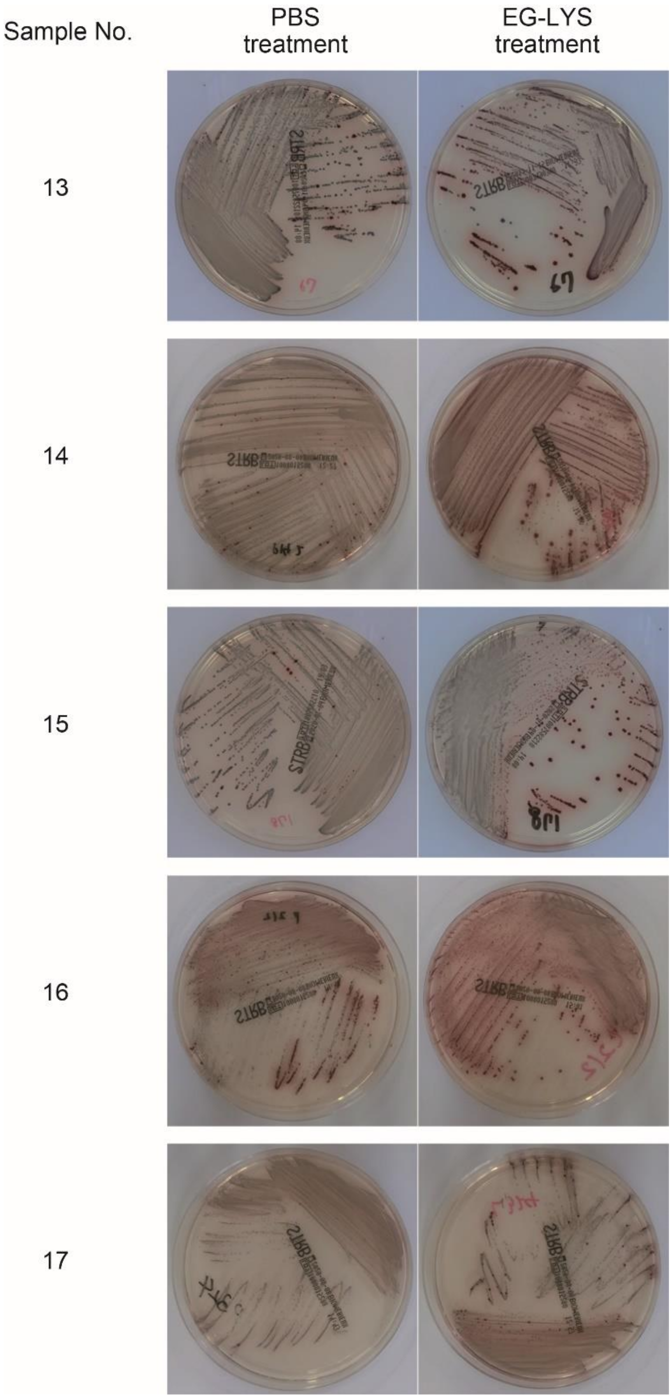


**Supplementary Fig. S3.** Photographs of chromogenic agars for GBS-positive samples with increased GBS detectability by EG-LYS treatment. These GBS-positive samples were designated as No. 13 to 17 in this study. Samples No. 13 to 17 showed thicker loads of GBS colonies on the chromogenic agars subcultured from the enriched broth in EG-LYS treatment than those in PBS treatment. Red colonies are GBS.

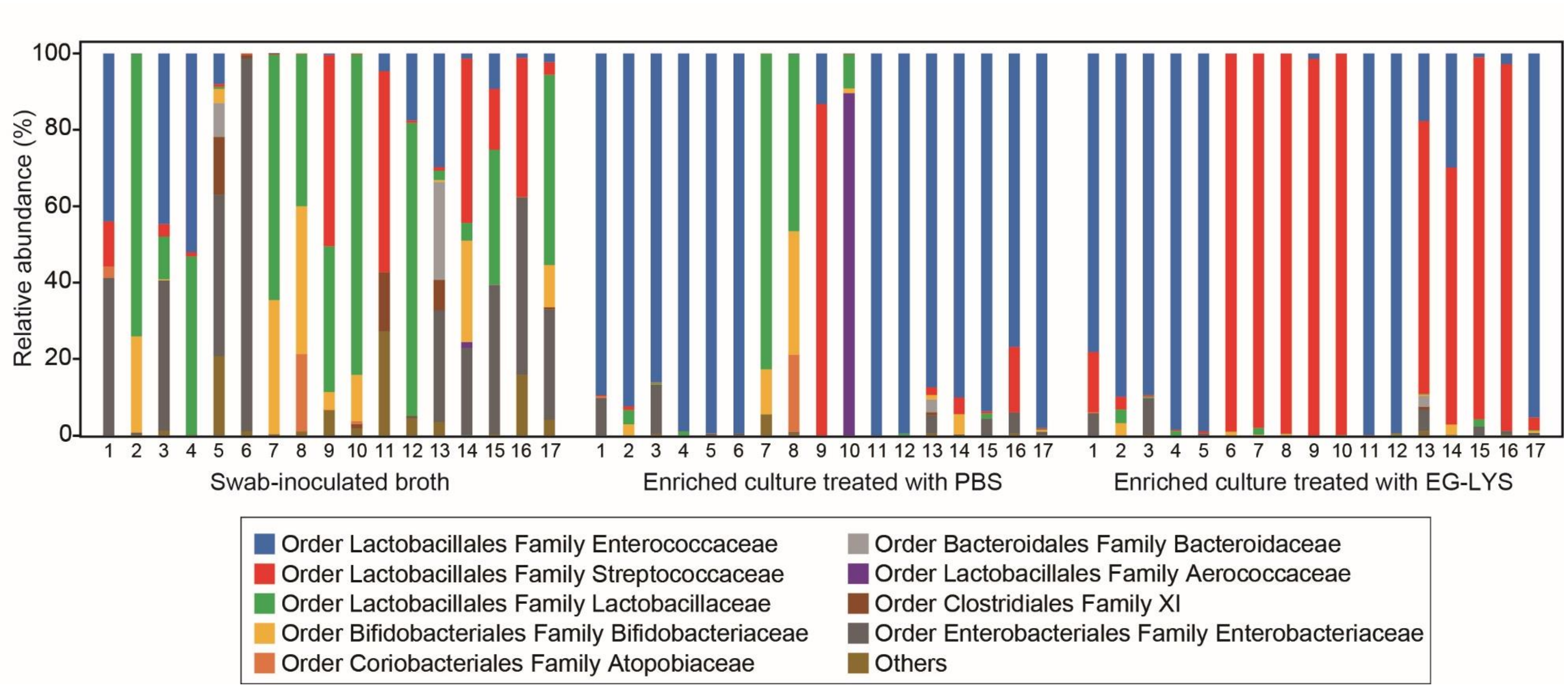


**Supplementary Fig. S4.** Microbial community bar plots at the family level of seventeen pairs of swab-resuspended broth and enriched cultures treated with PBS and EG-LYS. Relative abundances are shown in bar plots, and taxonomies are shown in different colors as described in the square below the plot. The taxa with relative abundances < 0.1% were assigned as "others". The sample number is shown under each bar plot. Samples 1 to 12 are the false-negative samples that showed GBS-positive results in only the enriched cultures treated with EG-LYS. Samples 13 to 17 are the GBS-positive samples that showed GBS-positive results in both enriched cultures treated with EG-LYS and PBS but showed thicker loads of GBS colonies from the enriched cultures treated with EG-LYS.

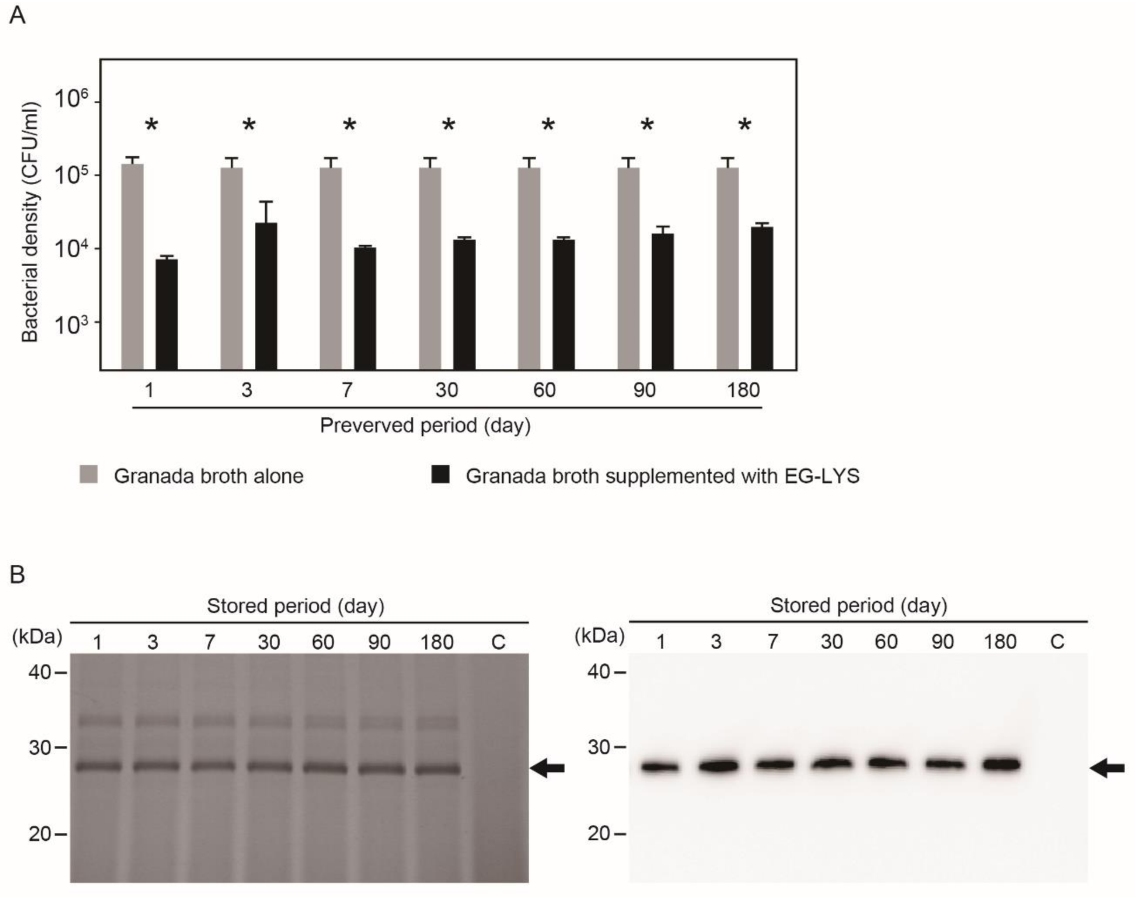


**Supplementary Fig. S5.** Stability of EG-LYS in Granada broth for 180 days. The Granada broths containing EG-LYS at 0.1 mg/ml were kept at 4 °C for 180 days. These stored broths were sampled over time. As a negative control, fresh Granada broth alone was used in both experiments. Immediately after sampling, small volumes of Granada broths were stored at −80 °C for the SDS-PAGE and western blot analyses. First, the *E. faecalis*-killing activity was examined. *E. faecalis* strain KUEF29 was grown until mid-log phase, then washed with PBS three times. The bacterial cells were suspended at 2.5 × $10^4$ CFU/ml in 10 ml of the Granada broth containing EG-LYS. After incubation at 20 °C for 1 h, the number of bacteria was measured. Second, 5 µl of the stored Granada broth containing EG-LYS was supplemented with an equal volume of ×2 sample buffer, and the proteins were separated on a 12.5% SDS-PAGE gel and western blotting was performed. The recombinant ORF60 in EG-LYS was detected using anti-6× His antibody on the western blot. (A) *E. faecalis*-killing activity analysis. The bacterial cell densities are shown as bar charts with error bars. The Granada broth alone and Granada broth supplemented with EG-LYS are shown in different colors, which are described below the bar charts. The experiments were repeated three times. Statistical analysis was performed

using the Student's T test. Statistical significance is denoted by an asterisk ($P < 0.005$). The Granada broth supplemented with EG-LYS showed significantly lower bacterial concentration than the Granada broth alone. Thus, the *E. faecalis*-killing activity remained stable for 180 days. (B) SDS-PAGE and western blot analyses. The images of SDS-PAGE (left) and western blot (right) are shown. On the top of each lane, the preserved period is shown. The label "C" is the negative control, which is Granada broth alone. On the leftmost, the molecular standards are shown. The arrows indicate the recombinant ORF60. The band thicknesses in the stored samples were not visually different from each other on the SDS-PAGE or the western blot images. Considering these results of *E. faecalis*-killing activity analysis together with SDS-PAGE and western blot analyses, the EG-LYS suspended in Granada broth was considered to be stable for 180 days.

Turbidity of pepetidoglycan and cells treated with “EG-LYS” or “PBS” at X min is put as

$$OD^{EG\text{-}LYS}_{595\ [x\ min]} \quad \text{or} \quad OD^{PBS}_{595\ [x\ min]} .$$

Turbidity change of peptidoglycan and cells treated with EG-LYS or PBS from X min to Y min (X < Y) is put as

$$\Delta OD^{EG\text{-}LYS}_{595\ [Y\text{-}X\ min]} = OD^{EG\text{-}LYS}_{595\ [X\ min]} - OD^{EG\text{-}LYS}_{595\ [Y\ min]} \quad \text{or}$$

$$\Delta OD^{PBS}_{595\ [Y\text{-}X\ min]} = OD^{PBS}_{595\ [X\ min]} - OD^{PBS}_{595\ [Y\ min]} .$$

Rate of turbidity change of peptidoglycan and cells treated with EG-LYS from X min to Y min (X < Y) is put as

$$\Delta OD^{EG\text{-}LYS\ -\ PBS}_{595\ [Y\text{-}X\ min]} = \Delta OD^{EG\text{-}LYS}_{595\ [Y\text{-}X\ min]} - \Delta OD^{PBS}_{595\ [Y\text{-}X\ min]} .$$

The quantity of EG-LYS is put as Z mg.

Reaction rate of EG-LYS treatment is put as

$$\Delta OD^{EG\text{-}LYS}_{595} = \Delta OD^{EG\text{-}LYS\ -\ PBS}_{595\ [Y\text{-}X\ min]} \ / \ Y\text{-}X\ min\ /\ Z\ mg .$$

In this study, X min, Y min and Z mg were put as 5 min, 15 min and 0.01 mg, respectively.
The reaction rate of EG-LYS, $\Delta OD^{EG\text{-}LYS}_{595}$, was calculated.

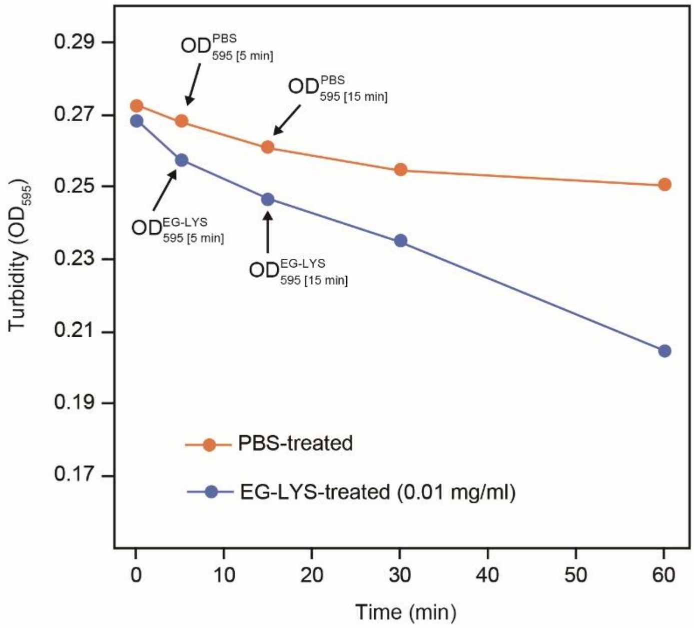


**Supplementary Fig. S6.** Calculation of EG-LYS reaction velocity.

The bacterial cell density in CFU/ml of "EG-LYS-treated" and "PBS-treated" bacterial culture after X h incubation is put as $CFU^{EG\text{-}LYS\text{-}treated}_{[X\ h]}$ or $CFU^{PBS\text{-}treated}_{[X\ h]}$, respectively.

The bacterial cell density change of "EG-LYS-treated" or "PBS-treated" from 0 h to X h is put as

$$\Delta log_{10}CFU^{EG\text{-}LYS\text{-}treated}_{[X\ h]} = log_{10}CFU^{EG\text{-}LYS\text{-}treated}_{[X\ h]} - log_{10}CFU^{PBS\text{-}treated}_{[0\ h]} \quad \text{or}$$

$$\Delta log_{10}CFU^{PBS\text{-}treated}_{[X\ h]} = log_{10}CFU^{PBS\text{-}treated}_{[X\ h]} - log_{10}CFU^{PBS\text{-}treated}_{[0\ h]} \ .$$

The inhibited bacterial cell density by EG-LYS treatment from 0 h to X h is put as

$$\Delta log_{10}CFU^{EG\text{-}LYS}_{[X\ h]} = \Delta log_{10}CFU^{PBS\text{-}treated}_{[0\ h]} - \Delta log_{10}CFU^{EG\text{-}LYS\text{-}treated}_{[X\ h]} \ .$$

The quantity of EG-LYS is put as Y mg.

The bacterial growth inhibitory efficiency by EG-LYS treatment is put as

$$\Delta log_{10}CFU^{EG\text{-}LYS}/h/mg = \Delta log_{10}CFU^{EG\text{-}LYS}_{[X\ h]} \ / \ X\ h \ / \ Y\ mg \ .$$

In this study, EG-LYS was treated at 0.01 mg/ml for 3-6 h (i.e., X h = 3-6 h, and Y mg = 0.01 mg), and the bacterial growth inhibitory efficiency, $\Delta log_{10}CFU^{EG\text{-}LYS}/h/mg$, was calculated.

**Supplementary Fig. S7.** Calculation method for bacterial growth inhibitory efficiency by EG-LYS treatment.
